# Genomic surveillance of *Pseudomonas aeruginosa* in the Philippines, 2013–2014

**DOI:** 10.5365/wpsar.2020.11.1.006

**Published:** 2021-04-28

**Authors:** Jeremiah Chilam, Silvia Argimón, Marilyn T. Limas, Melissa L. Masim, June M. Gayeta, Marietta L. Lagrada, Agnettah M. Olorosa, Victoria Cohen, Lara T. Hernandez, Benjamin Jeffrey, Khalil Abudahab, Charmian M. Hufano, Sonia B. Sia, Matthew T.G. Holden, John Stelling, David M. Aanensen, Celia C. Carlos, on behalf of the Philippines Antimicrobial Resistance Surveillance Program

**Affiliations:** a Antimicrobial Resistance Surveillance Reference Laboratory, Research Institute for Tropical Medicine, Muntinlupa, Philippines.; b Centre for Genomic Pathogen Surveillance, Wellcome Genome Campus, Hinxton, England, United Kingdom of Great Britain and Northern Ireland.; c Brigham and Women’s Hospital, Boston (MA), USA.; d University of St Andrews School of Medicine, St Andrews, Scotland, United Kingdom of Great Britain and Northern Ireland.; e Centre for Genomic Pathogen Surveillance, Big Data Institute, University of Oxford, Oxford, England, United Kingdom of Great Britain and Northern Ireland.; † These authors contributed equally to this work.; * These authors contributed equally to this work.

## Abstract

*Pseudomonas aeruginosa* is an opportunistic pathogen that often causes nosocomial infections resistant to treatment. Rates of antimicrobial resistance (AMR) are increasing, as are rates of multidrug-resistant (MDR) and possible extensively drug-resistant (XDR) infections. Our objective was to characterize the molecular epidemiology and AMR mechanisms of this pathogen.

We sequenced the whole genome for each of 176 *P. aeruginosa* isolates collected in the Philippines in 2013–2014; derived the multilocus sequence type (MLST), presence of AMR determinants and relatedness between isolates; and determined concordance between phenotypic and genotypic resistance.

Carbapenem resistance was associated with loss of function of the OprD porin and acquisition of the metallo-β-lactamase (MBL) gene *bla*^VIM^. Concordance between phenotypic and genotypic resistance was 93.27% overall for six antibiotics in three classes, but varied among aminoglycosides. The population of *P. aeruginosa* was diverse, with clonal expansions of XDR genomes belonging to MLSTs ST235, ST244, ST309 and ST773. We found evidence of persistence or reintroduction of the predominant clone ST235 in one hospital, and of transfer between hospitals.

Most of the ST235 genomes formed a distinct lineage from global genomes, thus raising the possibility that they may be unique to the Philippines. In addition, long-read sequencing of one representative XDR ST235 isolate identified an integron carrying multiple resistance genes (including *bla*^VIM-2^), with differences in gene composition and synteny from the *P. aeruginosa* class 1 integrons described previously.

The survey bridges the gap in genomic data from the Western Pacific Region and will be useful for ongoing surveillance; it also highlights the importance of curtailing the spread of ST235 within the Philippines.

*Pseudomonas aeruginosa* is an opportunistic pathogen that often causes nosocomial infections (e.g. pneumonia, bacteraemia and urinary tract infections), particularly in immunocompromised patients. (*1*) Eight Asian countries reported frequencies of isolation of *Pseudomonas* spp. of above 15% from hospital-acquired (HA) pneumonia cases, with the Philippines reporting *P. aeruginosa* as the most common etiological agent. (*2*) Also, Pseudomonas spp. were the second most common pathogen isolated from device-associated HA infections in a study of intensive care units in Philippine hospitals. (*3*)

*P. aeruginosa* infections are often resistant to treatment, (*4*) and carbapenem use has been strongly associated with resistance. (*1*) However, a study evaluating carbapenem restriction practices at a hospital in Manila found that 37% of the carbapenem prescriptions were non-compliant, highlighting challenges in antimicrobial stewardship. (*5*) Between 2010 and 2014, the Philippine Antimicrobial Resistance Surveillance Program (ARSP) reported increasing rates of resistance to antibiotics used to treat *P. aeruginosa* infections, such as carbapenems and extended-spectrum cephalosporins (**Fig. 1A-B**). In contrast, resistance to aminoglycosides and fluoroquinolones remained relatively stable or decreased slightly in the same period (**Fig. 1C**). The ARSP has also reported multidrug-resistant (MDR) rates of 21–23% and possible extensively drug-resistant (XDR) rates of 13–18% in recent years. (*6*-*8*)

**Figure 1A:**
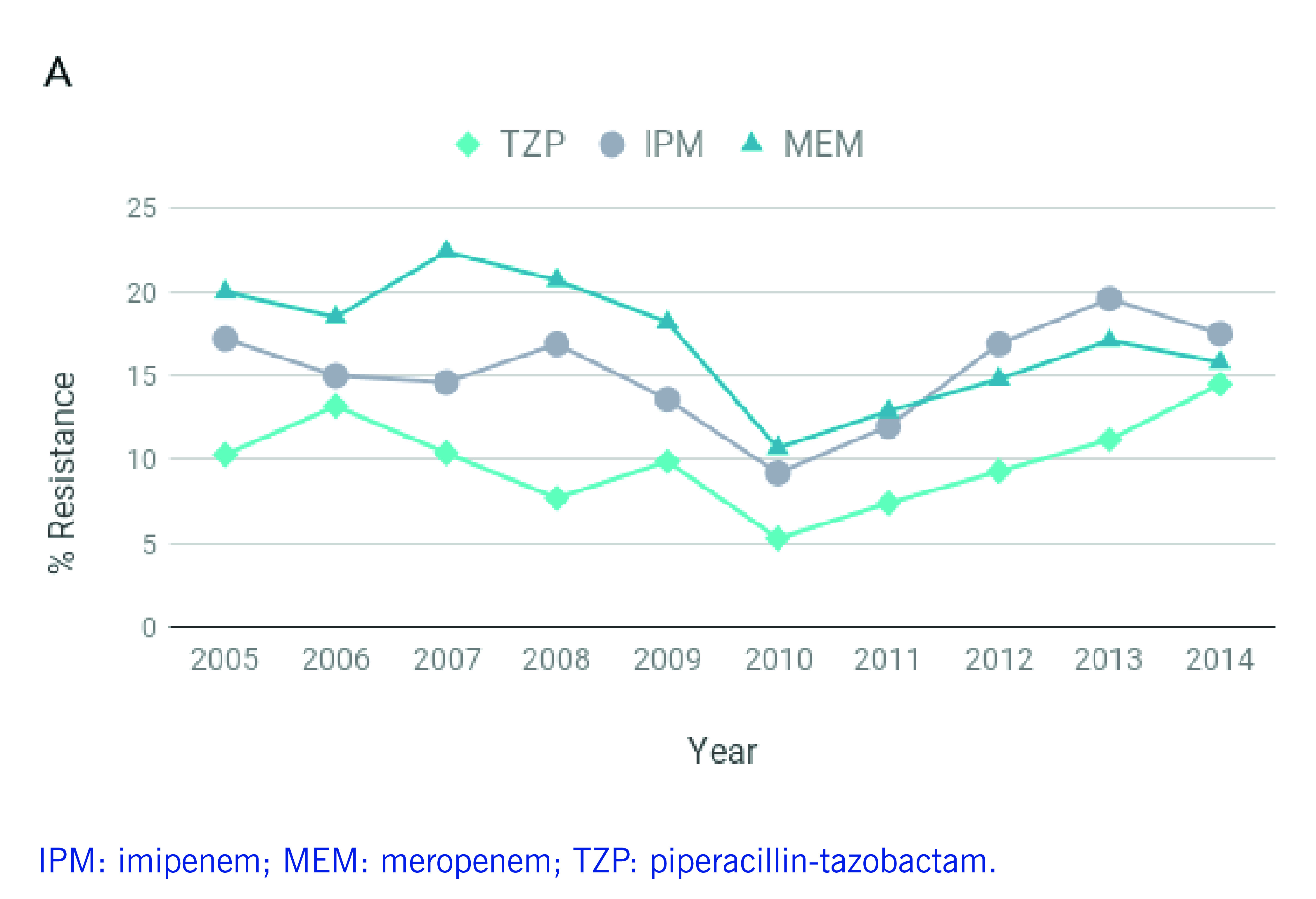
Annual resistance rates to nine antibiotics of P. aeruginosa isolates referred to the ARSP, 2005–December 2014

[**insert** Figure 1A]

**Figure 1B:**
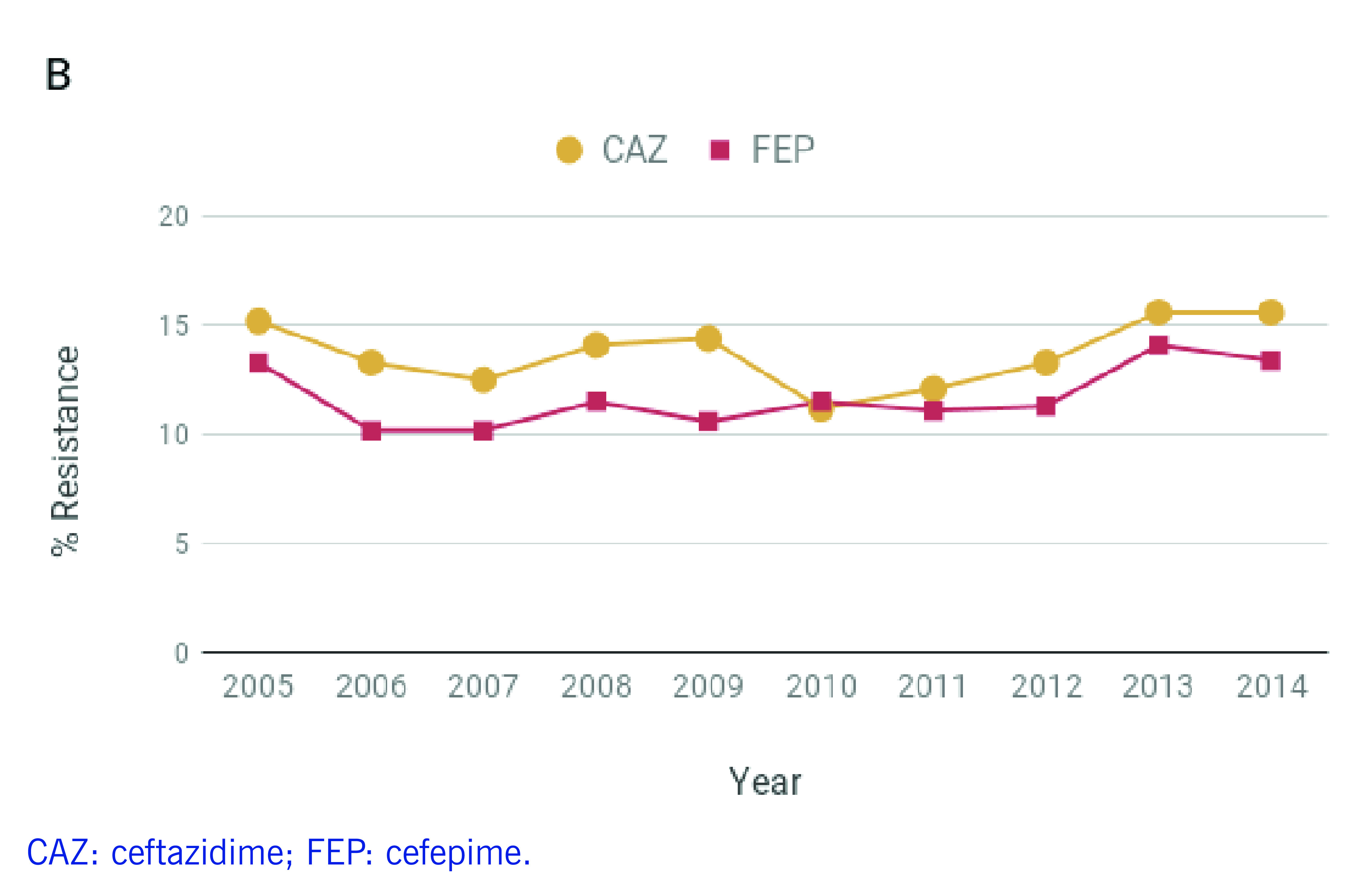
Annual resistance rates to nine antibiotics of P. aeruginosa isolates referred to the ARSP, 2005–December 2014

[**insert** Figure 1B]

**Figure 1C:**
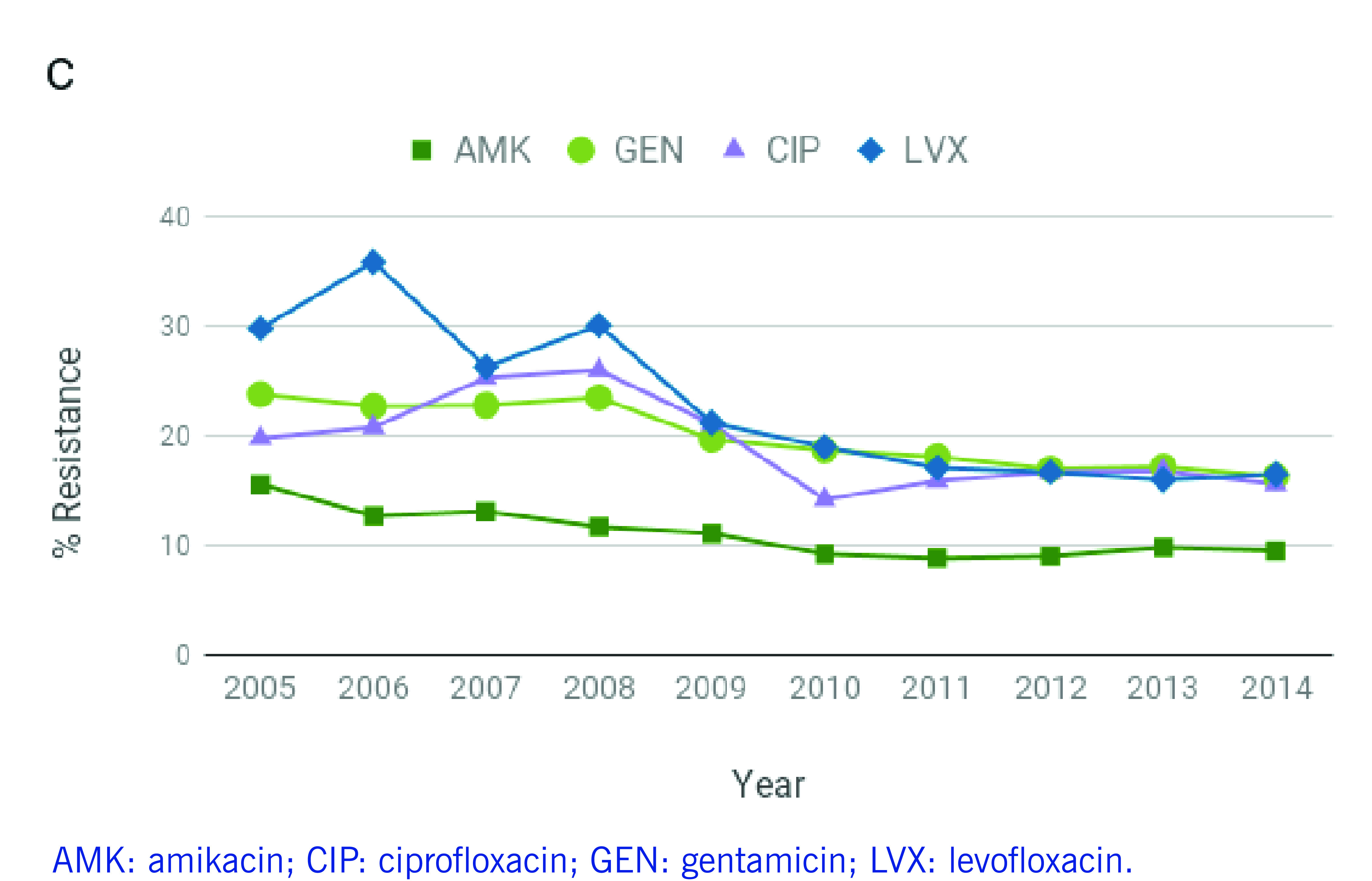
Annual resistance rates to nine antibiotics of P. aeruginosa isolates referred to the ARSP, 2005–December 2014

[**insert** Figure 1C]

The emergence of MDR *P. aeruginosa* with resistance to carbapenems, aminoglycosides and fluoroquinolones was followed by reports of isolates sensitive only to colistin (*9*) and, more recently, of colistin resistance in carbapenem non-susceptible isolates, (*10*) leaving few treatment options. These reports coincide with multilocus sequence type (MLST) ST235, (*9*-*11*) the predominant global epidemic clone. The metallo-β-lactamase (MBL) genes *bla*^VIM^ and *bla*^IMP^ – usually associated with integrons carrying multiple resistance determinants – have been identified in ST235 *P. aeruginosa* isolates from Asian countries. (*12*-*14*)

While the resistance rates and profiles of *P. aeruginosa* in the Philippines have been well characterized, (*15*, *16*) the molecular epidemiology and AMR mechanisms of this pathogen remain largely unknown. Whole-genome sequencing (WGS) can identify transmission patterns, AMR mechanisms and the source of HA infections. (*17*) In this study, we characterized the clonal relatedness and resistance determinants of *P. aeruginosa* isolates from the ARSP using WGS.

## Methods

### Bacterial isolates

A total of 7877 *P. aeruginosa* isolates were collected and tested for resistance by the ARSP from January 2013 to December 2014. Of the 443 and 283 isolates referred to the Antimicrobial Resistance Surveillance Reference Laboratory (ARSRL) for confirmation in 2013 and 2014, respectively, 179 isolates from 17 sentinel sites were selected for WGS, as previously described. (*18*) Briefly, 113 isolates of carbapenemase-producing *P. aeruginosa* were selected; also included were 66 available isolates that were susceptible to all antibiotics tested. We used a proxy definition for “infection origin,” whereby initial infection isolates collected in the community or on either of the first 2 days of hospitalization were categorized as community-acquired (CA), and isolates collected on hospital day 3 or later were categorized as hospital-acquired (HA). (*19*)

### Antimicrobial susceptibility testing (AST)

All *P. aeruginosa* isolates from this study were tested for susceptibility to nine antibiotics representing five classes: amikacin (AMK), ceftazidime (CAZ), ciprofloxacin (CIP), cefepime (FEP), gentamicin (GEN), imipenem (IPM), meropenem (MEM), tobramycin (TOB), and piperacillin-tazobactam (TZP) (**Table 1**). Antimicrobial susceptibility of the isolates was determined at ARSRL using the Kirby-Bauer disk diffusion method, and gradient methods such as E-Test (bioMérieux, Marcy-l’Étoile, France) and Vitek 2 Compact automated system (bioMérieux). To determine the resistance profile of the isolates, the zone of inhibition and minimum inhibitory concentration of antibiotics were interpreted according to guidelines from the Clinical and Laboratory Standard Institute (CLSI). (*20*) MDR phenotypes were classified according to standard definitions. (*21*)

**Table 1:** Total number of *P. aeruginosa* isolates analysed by the ARSP and referred to the ARSRL during 2013 and 2014, isolates submitted for WGS, and high-quality *P. aeruginosa* genomes obtained, discriminated by sentinel site and AMR profile

-	Number of isolates		
	2013	2014	Total
**Total ARSP**	3591	4286	7877
**Referred to ARSRL**	443	283	726
**Submitted for WGS**	89	90	179
**High-quality genomes**	87	89	176
By sentinel site *			
BGH	2	4	6
BRH	0	5	5
CMC	0	1	1
CVM	2	3	5
DMC	5	2	7
EVR	2	2	4
FEU	2	2	4
GMH	4	4	8
JLM	2	5	7
MMH	3	5	8
NKI	10	16	26
NMC	3	8	11
RMC	2	0	2
SLH	0	1	1
STU	5	4	9
VSM	32	16	48
By AMR profile **			
Susceptible	36	30	66
CAZ FEP IPM MEM TZP GEN TOB AMK CIP [XDR]	30	29	59
IPM MEM	7	9	16
CAZ FEP IPM MEM TZP GEN TOB CIP [XDR]	4	7	11
CAZ FEP IPM MEM TZP GEN TOB AMK	1	4	5
CIP	3	2	5
CAZ FEP IPM MEM TZP	1	2	3
IPM MEM TZP CIP	0	1	1
GEN TOB CIP	1	0	1
FEP TZP TOB CIP	0	1	1
CAZ FEP IPM MEM GEN TOB	1	0	1
IPM	1	0	1
CAZ FEP IPM MEM GEN TOB CIP	1	0	1
IPM MEM CIP	1	0	1
CAZ FEP GEN TOB AMK CIP	0	1	1
FEP IPM MEM GEN TOB CIP	0	1	1
CAZ	0	1	1
CAZ FEP TZP	0	1	1

* AMK: amikacin; AMR: antimicrobial resistance; ARSP: Antimicrobial Resistance Surveillance Program; ARSRL: Antimicrobial Resistance Surveillance Reference Laboratory; CAZ: ceftazidime; CIP: ciprofloxacin; FEP: cefepime; GEN: gentamicin; IPM: imipenem; MEM: meropenem; TOB: tobramycin; TZP: piperacillin-tazobactam; XDR: extensively drug resistant; WGS: whole-genome sequencing.

** BGH: Baguio General Hospital and Medical Center; BRH: Batangas Medical Center; CMC: Cotabato Regional and Medical Center; CVM: Cagayan Valley Medical Center; DMC: Southern Philippines Medical Center; EVR: Eastern Visayas Regional Medical Center; FEU: Far Eastern University – Nicanor Reyes Medical Foundation; GMH: Governor Celestino Gallares Memorial Hospital; JLM: Jose B. Lingad Memorial Regional Hospital; MMH: Corazon Locsin Montelibano Memorial Regional Hospital; NKI: National Kidney and Transplant Institute; NMC: Northern Mindanao Medical Center; RMC: Rizal Medical Center; SLH: San Lazaro Hospital; STU: University of Santo Tomas Hospital; VSM: Vicente Sotto Memorial Medical Center.

### DNA extraction and WGS

A total of 179 *P. aeruginosa* isolates were shipped to the Wellcome Trust Sanger Institute for WGS. DNA was extracted from a single colony of each isolate with the QIAamp 96 DNA QIAcube HT kit and a QIAcube HT (Qiagen, Hilden, Germany). DNA extracts were multiplexed and sequenced on the Illumina HiSeq platform (Illumina, CA, USA) with 100-bp paired-end reads. Isolate 13ARS-VSM740 was also sequenced with the PacBio RSII platform (Pacific Biosciences). Raw sequence data were deposited in the European Nucleotide Archive (ENA) under the study accession PRJEB17615. Run accessions for Illumina data are provided on the Microreact projects. The PacBio data were deposited under run accession ERR3284501.

### Bioinformatics analysis

Genome quality was evaluated based on metrics generated from assemblies, annotation files and the alignment of the isolates to the reference genome of *P. aeruginosa* strain LESB58 (accession FM209186), as previously described. (*18*) Assemblies were produced from short-read Illumina data (*18*) and from long-read PacBio data with the HGAP v4 pipeline (Pacific Biosciences). A total of 176 isolates yielded high-quality *P. aeruginosa* genomes and were included in this study.

We derived the MLST of the isolates from the whole genome sequences. The sequence types (ST) were determined from assemblies with Pathogenwatch (https://pathogen.watch/) and with MLSTcheck v1.007001, and from sequence reads with ARIBA (*22*) and the *P. aeruginosa* database hosted at PubMLST. (*23*) The MLST calls were curated, as previously described. (*18*) Integrons were detected in the genome assemblies with IntegronFinder. (*24*)

Evolutionary relationships between the 176 isolates were inferred from core single-nucleotide polymorphism (SNP). A core gene alignment was performed with Roary v3.11.3, using the mafft aligner option and minimum percentage identity for blastp of 90%. Evolutionary relationships between 169 isolates from groups 1 and 2 were inferred from SNPs by mapping the paired-end reads to the reference genomes of *P. aeruginosa* strains LESB58 (ST146, FM209186) or NCGM2_S1 (ST235, AP012280.1). (*18*) Mobile genetic elements (MGEs) were masked in the alignment of pseudogenomes with a script available at https://github.com/sanger-pathogens/remove_blocks_from_aln. For the phylogenetic analysis of ST235 genomes, recombination regions detected with Gubbins (*25*) were also removed. Alignments of SNPs were inferred with snp-sites v2.4.1, (*26*) and were used to compute pairwise SNP differences between isolates from different patients (minimum *n* = 3) belonging to the same or to different hospitals, using a script from https://github.com/simonrharris/pairwise_difference_count. Maximum likelihood phylogenetic trees were generated with RAxML, (*27*) based on the generalized time reversible (GTR) model with GAMMA method of correction for among-site rate variation and 100 bootstrap replications.

To contextualize the Philippine genomes, we downloaded, assembled and quality controlled global *P. aeruginosa* genomes with linked geographical and temporal information, collected mainly between 2007 and 2017, for which raw Illumina paired-end sequence data were available at the ENA. A tree of 904 genomes was inferred with FastTree (*28*) from an alignment of 549 126 SNP positions, obtained after mapping the reads to the complete genome of strain LESB58 and masking regions with MGEs. A tree of 96 global ST235 genomes was inferred with RAxML from an alignment of 1993 SNP sites obtained after mapping the genomes to the complete genome of strain NCGM2-S1, and masking MGEs and recombination regions.

Known AMR determinants were identified with ARIBA (*22*) and a curated database of known resistance genes and mutations, (*29*) the Comprehensive Antibiotic Resistance Database, (*30*) and a custom database of mutations in the quinolone resistance-determining region of the *gyrA/B* and *parC/E* genes described for *P. aeruginosa*. (*4*) The output for the porin gene *oprD* was inspected to detect loss-of-function mutations. The *oprD* sequences were extracted from the whole-genome draft assemblies with blastn, using the oprD sequence from strain PAO1 (accession NC_002516.2, genome positions 1043982–1045314) as a query, then translated in silico to inspect the integrity of the coding frames. A 444 or 442 amino-acid protein that included a START and a STOP codon was considered functional.

The genomic predictions of AMR derived from the presence of known AMR genes and mutations (test) were compared with the phenotypic results (reference), and concordance was computed for each of six antibiotics (1056 total comparisons). Isolates with either a resistant or an intermediate phenotype were considered non-susceptible. An isolate with the same outcome for both the test and reference (i.e. both susceptible or both non-susceptible) was counted as a concordant isolate. Concordance was the number of concordant isolates as a percentage of the total number of isolates assessed.

All project data, including inferred phylogenies, AMR predictions and metadata were made available through Microreact.

### Ethics statement

Ethical approval is not applicable. This study uses archived bacterial samples processed by the ARSP. No identifiable data were used in this study.

## Results

### Demographic and clinical characteristics of the *P. aeruginosa* isolates

Of the 179 *P. aeruginosa* isolates submitted for WGS, 176 passed quality control and were confirmed in silico as *P. aeruginosa* (**Table 2**). Patients were aged from under 1 to 96 years, with 27% (*n* = 47) of the isolates from patients aged 65 years or older. Fifty-eight per cent (*n* = 102) of the isolates were from HA infections. In terms of specimen type, 53% (*n* = 94) of isolates were from respiratory samples (tracheal aspirates and sputum).

**Table 2:** Demographic and clinical characteristics of 176 *P. aeruginosa* isolates^a^

Characteristic	No. isolates
**Sex**	
Male	119
Female	57
**Age (in years)**	
< 1	12
1–4	6
5–14	7
15–24	14
25–34	5
35–44	17
45–54	29
55–64	34
65–80	36
^3^ 81	11
Age unknown	5
**Patient type**	
Inpatient	159
Outpatient	17
**Specimen origin**	
Community-acquired	74
Hospital-acquired	102
**Submitted as**	
Carbapenem non-susceptible	100
Resistant to at least 1 antibiotic other than carbapenems	10
Susceptible	66
**Specimen type**	
Abdominal fluid*	1
Abscess	1
Blood*	21
Bronchial	1
Catheter	2
Cerebrospinal fluid*	3
Cornea	2
Dialysis fluid*	1
Drainage	1
Fluid	3
Inguinal	1
Other	1
Pleural fluid*	1
Sputum	31
Tissue	5
Tracheal	1
Tracheal aspirate	63
Urine	12
Wound	25

^a^ Invasive isolates were considered as those obtained from specimen types marked with an asterisk (*).

### Concordance between phenotypic and genotypic AMR

Isolates were tested for susceptibility to nine antibiotics representing five classes (**Fig. 1A-C**, **Table 3**). Most isolates were non-susceptible to carbapenems (*n* = 100), 10 isolates were susceptible to carbapenems but resistant to other antibiotics, and 66 isolates were susceptible to all nine antibiotics (**Table 1**). CA infections were more frequently associated with susceptible isolates and HA infections with resistant isolates (**Fig. 1D**, two-tailed Fisher’s exact test *P* = 0.000002).

**Figure 1D:**
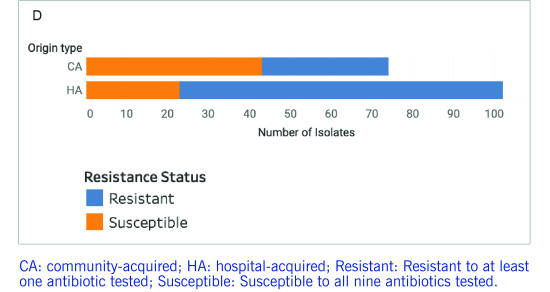
Association between resistance and the origin of infection for 176 P. aeruginosa isolates sequenced in this study

[**insert** Figure 1D]

**Table 3:** Comparison of genomic predictions of antibiotic resistance with laboratory susceptibility testing at the ARSRL

Antibiotic class	Antibiotic	Isolates tested	Resistant isolates (AST)	False positive	False negative	Concordance (%)	Acquired resistance mechanisms
Carbapenem	Imipenem	176	100	1	4	97.16	*^bla^*VIM-2^,^ *^bla^*VIM-6^,^ *^bla^*NDM-1^,^
	Meropenem	176	99	2	4	96.59	*^bla^*IMP-26^, OprD loss-of-func^tion (*oprD* interrupted, fragmented, or missing, presence of premature STOP, START codon missing), NalC/D loss-of-function (*nalC* missing, NalC_G71E, S209R, A186T, NalD_S32N)
Aminoglycoside	Gentamicin	176	77	0	34	80.68	AAC(3)-IIe, AAC(6’)-31, AAC(6’)-IIa, ANT(2”)-Ia
	Tobramycin	176	78	2	3	97.16	AAC(3)-IIe, AAC(6’)-31, AAC(6’)-Ib, AAC(6’)-Ib-cr, AAC(6’)-IIa, ANT(2”)-Ia
	Amikacin	176	61	14	4	89.77	AAC(6’)-31, AAC(6’)-Ib, AAC(6’)-IIa, APH(3¢)-VI
Fluoroquinolone	Ciprofloxacin	176	82	5	12	93.75	*qnrD, qnrVC*, AAC(6')-Ib-cr, *GyrA*_D87N, D87Y, T83I, *GyrB*_E468D, S466F, *ParC*_S87L

AST: antimicrobial susceptibility testing.

Of the 18 isolates resistant to imipenem and meropenem but not to other β-lactam antibiotics, 17 carried both loss-of-function disruptions in the OprD porin, and disruptions or known non-synonymous mutations in the NalC (A186T, G71E, S209R) and/or NalD (S32N) regulators of the MexAB-OprM multidrug efflux pump, suggesting that their resistance is due to a combination of reduced influx and increased efflux of the carbapenem antibiotics (**Fig. 1E**). Among the 81 carbapenem-resistant isolates that were also resistant to third-generation cephalosporin ceftazidime and/or fourth-generation cephalosporin cefepime, 67 isolates carried acquired MBL genes *bla*^VIM-2^ (*n* = 61 genomes), *bla*^VIM-6^ (*n* = 1), *bla*^IMP-26^ (*n* = 4) or *bla*^NDM-1^ (*n* = 1), while five carried disrupted *oprD* genes plus acquired extended-spectrum β-lactamase (ESBL) genes *bla*^PER-1^ (*n* = 3), *bla*^CTX-M-15^ (*n* = 1) or AmpC-like gene *bla*^DHA-1^ (*n* = 1). The remaining eight isolates harboured other β-lactamase genes, but their carbapenem-resistance mechanisms remain uncharacterized. Of the 76 isolates susceptible to carbapenems, 75 carried either a full-length OprD porin (444 amino acids) without any known mutations, or a 442 amino acid-long OprD protein with an intact reading frame, while one isolate was missing the STOP codon in the *oprD* gene.

**Figure 1E:**
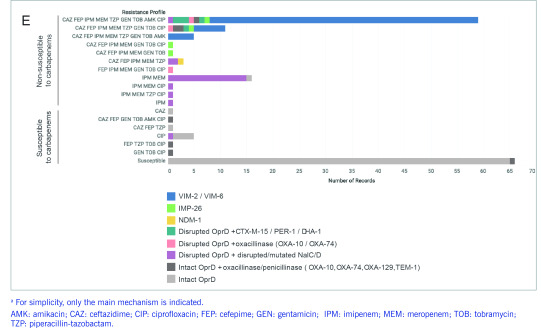
Mechanisms of resistance to carbapenems and other β-lactam antibiotics identified in the genomes of 176 isolates grouped by their resistance profile^a^

[**insert** Figure 1E]

The overall phenotypic and genotypic concordance was 93.27% for the six antibiotics analysed (**Table 3**). The concordance was above 96% for carbapenems.

### Genotypic findings

#### In silico genotyping

A total of 79 STs were identified (**Table 4**), with 27.8% (*n* = 49) belonging to ST235, followed by ST309 (5.7%, *n* = 10), ST244 and ST773 (5.1% each, *n* = 9). The majority of the STs (79.7%, *n* = 63) were singletons (represented by only one genome), most of which (*n* = 42) were contributed by the susceptible isolates. Indeed, the resistant isolates (36 STs, *n* = 110) exhibited less clonal diversity than the susceptible isolates (56 STs, *n* = 66). ST235 represented 43.6% (*n* = 48) of the resistant isolates but only 1.5% (*n* = 1) of the susceptible isolates, and was predominantly a nosocomial clone in the Philippines (36 HA vs 13 CA isolates), spread across 13 hospitals.

**Table 4:** Distribution of isolates, sequence types (STs), resistance profiles and acquired resistance mechanisms across the 17 sentinel sites^a^

Laboratory	No. of Isolates	No. of STs	Prevalent ST (no. of isolates)	Resistance profiles	Acquired resistance determinants
BGH	6	5	309 (2)	Susceptible (2) CAZ FEP IPM MEM TZP GEN TOB CIP (2) CAZ FEP IPM MEM TZP GEN TOB AMK CIP (1) IPM MEM (1)	NalC/D LOF (2) IMP-26, AAC(6')-Ib, QnrVC1, OprD LOF, NalC/D LOF (1) VIM-2, AAC(6')-Ib4, ANT(3”)-Ia, GyrA.D87Y, OprD LOF, NalC/D LOF (1) VIM-2, AAC(6')-Ib4, ANT(3”)-Ia, QnrVC1, GyrA.T83I, OprD LOF, NalC/D LOF (1) OprD LOF, NalC/D LOF (1)
BRH	5	3	235 (2)	CAZ FEP IPM MEM TZP GEN TOB AMK CIP (3) Susceptible (2)	AAC(6')-31, AAC(6')-Il, ANT(3”)-Ia, APH(3¢)-VI, GyrA.T83I, ParC.S87L, OprD LOF, NalC/D LOF (2) VIM-2, AAC(6')-Ib4, APH(3¢)-Ia, QnrVC1, NalC/D LOF (1) NalC/D LOF (2)
CMC	1	1	1121	CAZ FEP IPM MEM TZP GEN TOB AMK CIP (1)	ANT(2”)-Ia, ANT(3”)-Ia, QnrVC1, NalC/D LOF (1)
CVM	5	3	235 (3)	CAZ FEP IPM MEM TZP GEN TOB AMK CIP (2) Susceptible (2) CAZ FEP IPM MEM TZP GEN TOB CIP (1)	VIM-2, AAC(6')-Ib4, ANT(2”)-Ia, ANT(3”)-Ia, GyrA.T83I, ParC.S87L, OprD LOF, NalC/D LOF (2) NalC/D LOF (2) VIM-2, AAC(6')-Ib4, ANT(2”)-Ia, ANT(3”)-Ia, GyrA.T83I, ParC.S87L, OprD LOF, NalC/D LOF (1)
DMC	7	7	9, 463, 381, 244, 639, 303, 357 (1)	Susceptible (3) CAZ FEP IPM MEM TZP GEN TOB AMK CIP (2) IPM MEM (2)	NalC/D LOF (2) None (1) ANT(2”)-Ia, ANT(3”)-Ia, APH(3¢)-VI, QnrVC1, gyrB.S466F, OprD LOF, NalC/D LOF (1) IMP-26, AAC(6')-Ib4, ANT(2”)-Ia, ANT(3”)-Ia, QnrVC1, OprD LOF, NalC/D LOF (1) OprD LOF, NalC/D LOF (2)
EVR	4	4	1966~, 1978, 235, 1 823 (1)	Susceptible (2) CIP (1) CAZ FEP IPM MEM TZP GEN TOB CIP (1)	NalC/D LOF (2) NalC/D LOF (1) VIM-2, AAC(6')-Ib4, APH(3¢)-Ia, NalC/D LOF (1)
FEU	4	2	235 (3)	CAZ FEP IPM MEM TZP GEN TOB AMK CIP (2) IPM MEM CIP (1) Susceptible (1)	VIM-2, AAC(6')-Ib4, ANT(3”)-Ia, GyrA.T83I, ParC.S87L, OprD LOF, NalC/D LOF (2) GyrA.T83I, ParC.S87L, OprD LOF, NalC/D LOF (1) NalC/D LOF (1)
GMH	8	3	313(4)	CAZ FEP IPM MEM TZP GEN TOB AMK (4) CAZ FEP IPM MEM TZP GEN TOB AMK CIP (4)	VIM-2, AAC(6')-Ib4, NalC/D LOF (4) VIM-2, AAC(6')-Ib4, ANT(2”)-Ia, ANT(3”)-Ia, GyrA.T83I, ParC.S87L, OprD LOF, NalC/D LOF (2) VIM-2, AAC(6')-Ib4, ANT(2”)-Ia, ANT(3”)-Ia, GyrA.T83I, ParC.S87L, NalC/D LOF (1) VIM-6, AAC(6')-Ib4, GyrA.T83I, ParC.S87L, NalC/D LOF (1)
JLM	7	7	244, 1 597, 381, 261, 2 330, 309, 316 (1)	Susceptible (6) FEP TZP TOB CIP (1)	NalC/D LOF (5) None (1) AAC(6')-Ib-cr, GyrA.T83I, ParC.S87L, NalC/D LOF (1)
MAR	24	20	357 (3)	Susceptible (14) IPM MEM (6) CAZ FEP IPM MEM TZP (2) CAZ FEP IPM MEM TZP GEN TOB CIP (1) GEN TOB CIP (1)	NalC/D LOF (14) OprD LOF, NalC/D LOF (4) AAC(6')-Ib, OprD LOF, NalC/D LOF (1) gyrB.E468D, OprD LOF, NalC/D LOF (1) OprD LOF, NalC/D LOF (2) ANT(2”)-Ia, ANT(3”)-Ia, GyrA.T83I, ParC.S87L, NalC/D LOF (1) QnrVC1, NalC/D LOF (1)
MMH	8	5	272~(3)	CAZ FEP IPM MEM TZP GEN TOB AMK CIP (2) CAZ FEP IPM MEM GEN TOB (1) CAZ FEP IPM MEM GEN TOB CIP (1) CAZ FEP IPM MEM TZP (1) CAZ FEP IPM MEM TZP GEN TOB AMK (1) CAZ FEP IPM MEM TZP GEN TOB CIP (1) IPM MEM TZP CIP (1)	VIM-2, AAC(3)-IIe, APH(3¢)-Ia, APH(3¢)-VI, OprD LOF, NalC/D LOF (2) IMP-26, AAC(6')-Ib4, APH(3¢)-Ia, APH(3¢)-VI, NalC/D LOF (1) IMP-26, AAC(6')-Ib4, APH(3¢)-Ia, APH(3¢)-VI, NalC/D LOF (1) NDM-1, ANT(3”)-Ia, APH(3¢)-VI, OprD LOF, NalC/D LOF (1) VIM-2, AAC(3)-Iie, APH(3¢)-Ia, APH(3¢)-VI, NalC/D LOF (1) AAC(3)-IIe, AAC(6')-Ib4, ANT(3”)-Ia, APH(3¢)-Ia, GyrA.T83I, ParC.S87L, OprD LOF, NalC/D LOF (1) gyrB.E468D, OprD LOF, NalC/D LOF (1)
NKI	26	21	235 (5)	Susceptible (15) CAZ FEP IPM MEM TZP GEN TOB AMK CIP (3) CIP (3) CAZ FEP GEN TOB AMK CIP (1) CAZ FEP TZP (1) FEP IPM MEM GEN TOB CIP (1) IPM (1) IPM MEM (1)	NalC/D LOF (14) None (1) AAC(6')-31, AAC(6')-Il, ANT(3”)-Ia, APH(3¢)-VI, GyrA.T83I, ParC.S87L, OprD LOF, NalC/D LOF (1) VIM-2, AAC(6')-Ib4, ANT(2”)-Ia, ANT(3”)-Ia, GyrA.T83I, VIM-2, AAC(6')-Ib4, ANT(3”)-Ia, QnrVC1, NalC/D LOF (1) GyrA.D87N, OprD LOF, NalC/D LOF (1) NalC/D LOF (1) QnrVC1, NalC/D LOF (1) GyrA.T83I, ParC.S87L, NalC/D LOF (1) NalC/D LOF (1) ANT(2”)-Ia, ANT(3”)-Ia, GyrA.T83I, ParC.S87L, OprD LOF, NalC/D LOF (1) OprD LOF, NalC/D LOF (1) ANT(3”)-Ia, OprD LOF, NalC/D LOF (1)
NMC	11	6	244 (6)	CAZ FEP IPM MEM TZP GEN TOB AMK CIP (6) Susceptible (5)	VIM-2, AAC(6')-Ib4, ANT(3”)-Ia, GyrA.T83I, ParC.S87L, OprD LOF, NalC/D LOF (4) AAC(6')-Iia, ANT(2”)-Ia, ANT(3”)-Ia, GyrA.T83I, ParC.S87L, OprD LOF, NalC/D LOF (1) VIM-2, AAC(6')-Ib4, QnrVC1, GyrA.T83I, ParC.S87L, OprD LOF, NalC/D LOF (1) NalC/D LOF (4) None (1)
RMC	2	2	1632, 235 (1)	CIP (1) Susceptible (1)	GyrA.T83I, ParC.S87L, NalC/D LOF (1) NalC/D LOF (1)
SLH	1	1	235	CAZ FEP IPM MEM TZP GEN TOB CIP (1)	AAC(6')-Ib4, ANT(3”)-Ia, APH(3¢)-Ia, GyrA.T83I, ParC.S87L, OprD LOF, NalC/D LOF (1)
STU	9	6	309 (3)	CAZ FEP IPM MEM TZP GEN TOB AMK CIP (3) IPM MEM (3) CAZ FEP IPM MEM TZP GEN TOB CIP (2) Susceptible (1)	VIM-2, AAC(6')-Ib4, ANT(3”)-Ia, QnrVC1, GyrA.T83I, OprD LOF, NalC/D LOF (3) OprD LOF, NalC/D LOF (2) NalC/D LOF (1) VIM-2, AAC(6')-Ib4, ANT(2”)-Ia, ANT(3”)-Ia, GyrA.T83I, ParC.S87L, NalC/D LOF (1) VIM-2, AAC(6')-Ib4, ANT(2”)-Ia, ANT(3”)-Ia, GyrA.T83I, ParC.S87L, OprD LOF, NalC/D LOF (1) None (1)
VSM	48	16	235 (24)	CAZ FEP IPM MEM TZP GEN TOB AMK CIP (30) Susceptible (12) IPM MEM (3) CAZ FEP IPM MEM TZP GEN TOB CIP (2) CAZ (1)	VIM-2, AAC(6')-Ib4, ANT(2”)-Ia, ANT(3”)-Ia, GyrA.T83I, ParC.S87L, OprD LOF, NalC/D LOF (9) VIM-2, AAC(6')-Ib4, ANT(2”)-Ia, ANT(3”)-Ia, QnrVC1, GyrA.T83I, ParC.S87L, OprD LOF, NalC/D LOF (8) VIM-2, AAC(6')-Ib4, ANT(2”)-Ia, ANT(3”)-Ia, QnrVC1, GyrA.T83I, ParC.S87L, NalC/D LOF (3) VIM-2, AAC(6')-Ib4, APH(3¢)-Ia, QnrVC1, NalC/D LOF (3) VIM-2, AAC(6')-Ib4, OprD LOF, NalC/D LOF (3) VIM-2, AAC(6')-Ib4, ANT(2”)-Ia, ANT(3”)-Ia, GyrA.T83I, ParC.S87L, NalC/D LOF (2) VIM-2, AAC(6')-Ib4, QnrVC1, OprD LOF, NalC/D LOF (1) gyrB.S466F, OprD LOF, NalC/D LOF (1) NalC/D LOF (12) OprD LOF, NalC/D LOF (3) VIM-2, AAC(6')-Ib4, ANT(2”)-Ia, ANT(3”)-Ia, QnrVC1, GyrA.T83I, ParC.S87L, NalC/D LOF (1) ANT(3”)-Ia, GyrA.T83I, ParC.S87L, NalC/D LOF (1) ANT(3”)-Ia, NalC/D LOF

LOF: loss-of-function

#### Population structure of P. aeruginosa in the Philippines

The phylogenetic tree of 176 genomes from the Philippines comprises three major groups, (*31*) group 1 (*n* = 64) including PA14, group 2 (*n* = 105) including PAO1 and the more distantly related group 3 (*n* = 7) including PA7 (**Fig. 2A**). All three groups included carbapenem-resistant isolates and susceptible isolates, though most isolates in group 2 were susceptible (*n* = 39, 60.9%) and most in group 1 were resistant (*n* = 75, 71.4%, **Fig. 2B**).

**Figure 2A:**
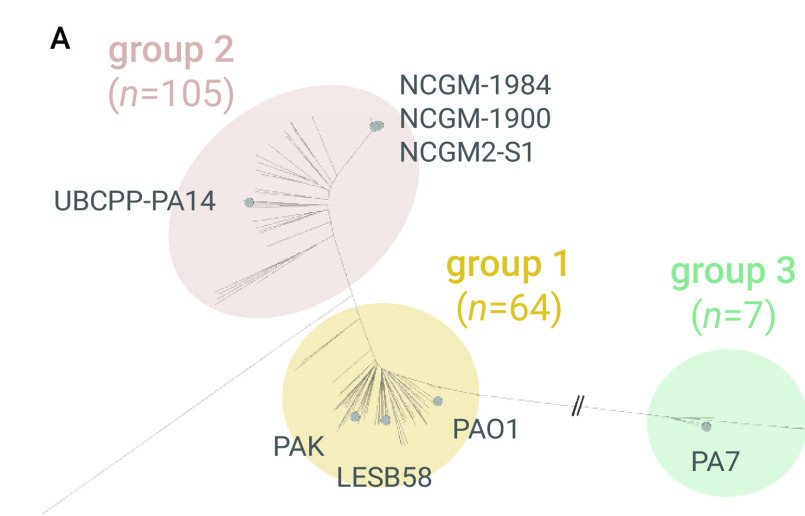
Genomic surveillance of P. aeruginosa from the Philippines, 2013–2014^a^

[**insert** Figure 2A]

**Figure 2B:**
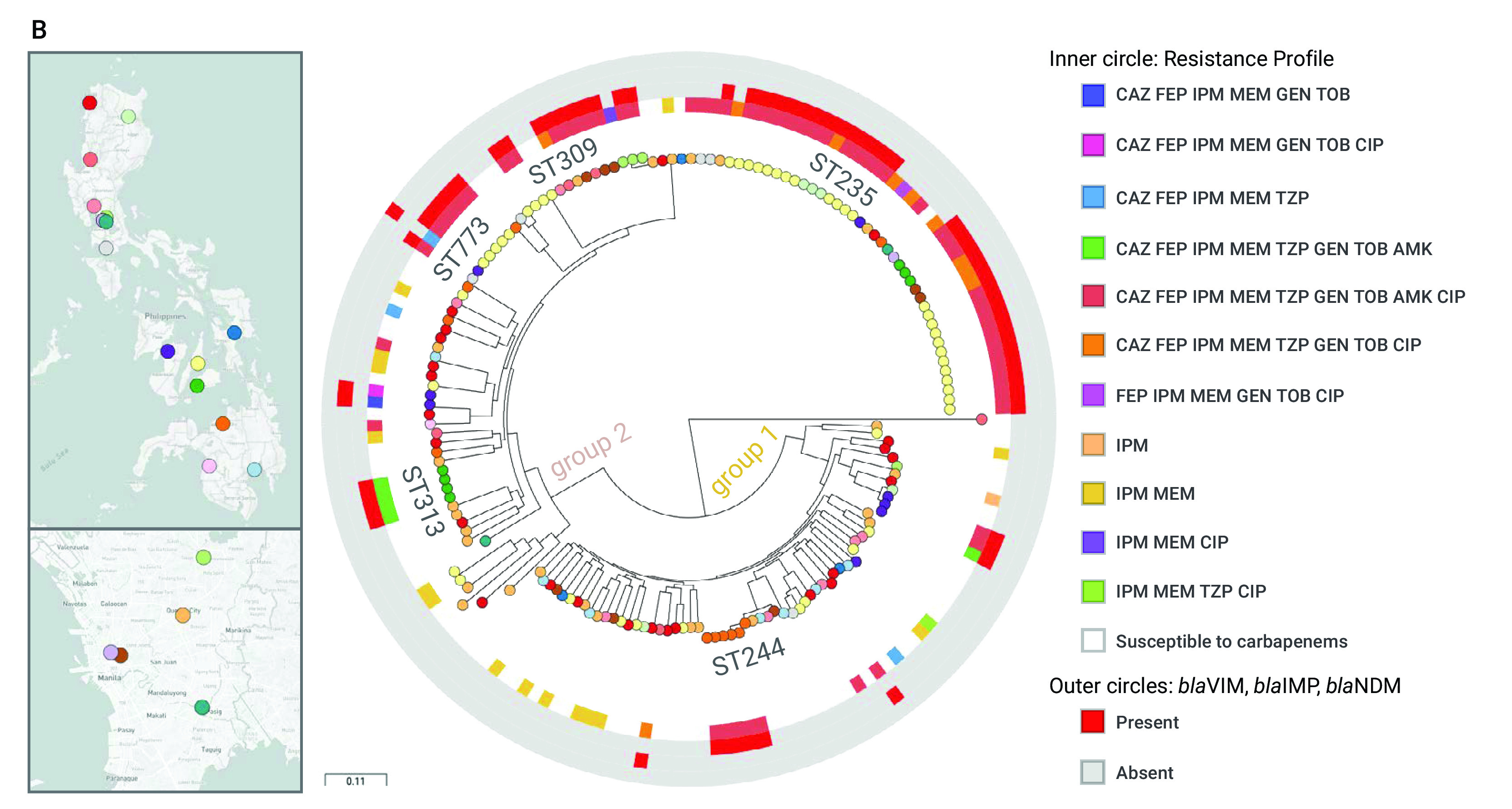
Genomic surveillance of P. aeruginosa from the Philippines, 2013–2014^a^

[**insert** Figure 2B]

The population of *P. aeruginosa* comprises a limited number of widespread clones selected from a diverse pool of rare, unrelated genotypes that recombine at high frequency. (*32*) A phylogenetic tree of 169 genomes from groups 1 and 2 showed that the clonal expansions were mostly within the major group 1 – represented by ST235, ST309, ST773 and ST313 (**Fig. 2B**) – found across multiple hospitals and resistant to multiple antibiotics. Most of the XDR isolates (*n* = 61, 87%) were found in ST235, ST244, ST309 and ST773, and most (*n* = 44, 62.8%) carried bla^VIM^ (an MBL that can degrade all anti-pseudomonal β-lactamases except for aztreonam), (*1*) AAC(6’)-Ib (an aminoglycoside acetyltransferase conferring resistance to tobramycin and amikacin), and the non-synonymous mutation T83I on GyrA associated with resistance to fluoroquinolones.

The higher prevalence of ST235 prompted us to look further at this clone. The phylogenetic tree of 49 ST235 isolates comprised two distinct clades with different geographic distribution (**Fig. 2C**). Clade I (*n* = 10) was represented in five hospitals in the Luzon (north) and Visayas (central) island groups, while clade II (*n* = 39) was represented in 10 hospitals from north to south of the country. The phylogeographic structure of the tree and the relatedness between genomes showed evidence of dissemination of ST235 between hospitals. Within clade Ib (**Fig. 2C**), one genome from hospital NKI differed from two genomes from hospital BRH by seven and eight SNPs, respectively. Within clade IIb (**Fig. 2C**), the genetic differences between isolates from the same hospital (mean pairwise SNP differences 36.41 ± 20.84, range 0–64) were not significantly different to those between isolates from different hospitals (mean 45.36 ± 8.12, range 29–61, Mann–Whitney U test z-score = –1.49145, *P* = 0.13622). The close relationships and the common repertoire of resistance genes between isolates from different hospitals support inter-hospital transmission.

The genomes from the hospital VSM (*n* = 24) formed at least three clusters within clade IIb, two of which exhibited discrete temporal distribution (VSM-2 and VSM-3, **Fig. 2C**), suggesting that they could represent hospital outbreaks. In agreement with this, the genomes from different patients within clade VSM-3 differed by an average of 11.55 pairwise SNPs (range 0–24). We also identified isolates within VSM-3 that were collected nine or more months apart (**Fig. 2C**), suggesting that ST235 can either persist in or be reintroduced to the hospital environment.

**Figure 2C:**
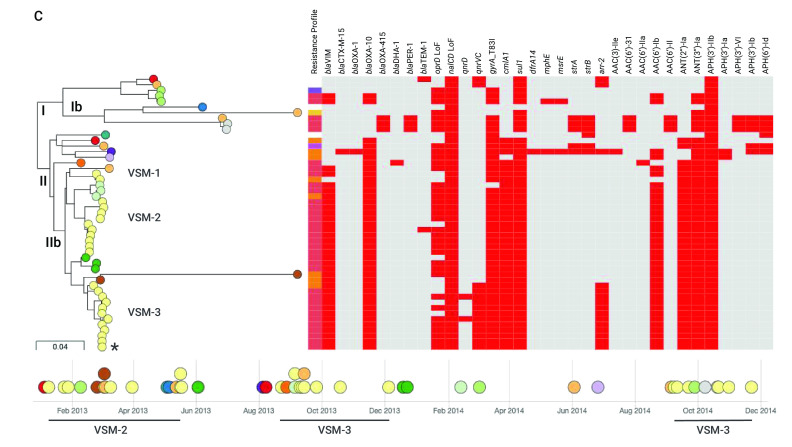
Genomic surveillance of P. aeruginosa from the Philippines, 2013–2014^a^

[**insert** Figure 2C]

The distribution of acquired resistance genes and mutations showed that resistance determinants differed between clades I and II, with patterns that were consistent with the acquisition of multiple genes simultaneously by mobile genetic elements. Long-read sequencing of isolate 14ARS-VSM0870, representative of the XDR resistant profile CAZ FEP IPM MEM TZP GEN TOB AMK CIP (marked with an asterisk on  **Fig. 2C**), revealed the acquisition of *bla*^VIM-2^, *bla*^OXA-10^, *catB3*, *aadA1* (ANT(3”)-Ia) and *acc(6’)-Ib* within a class 1 integron integrated in the chromosome at position  977 774 (**Fig. 2D**). The ciprofloxacin resistance gene *qnrVC* and the rifampin-resistance gene *arr-2* were located on a different class 1 integron elsewhere in the genome.

**Figure 2D:**
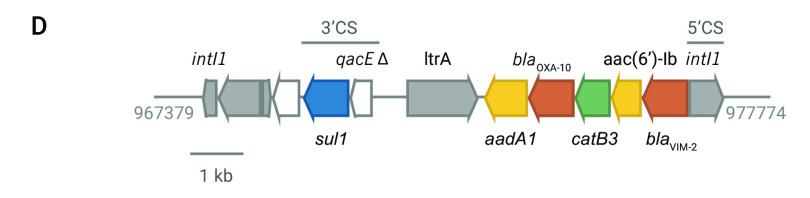
Genomic surveillance of P. aeruginosa from the Philippines, 2013–2014^a^

[**insert** Figure 2D]

#### P. aeruginosa from the Philippines in the global context

We placed the genomes from our retrospective collection in the global context of 904 contemporary  *P. aeruginosa* public genomes. This collection of public genomes represented 17 countries and 178 STs, with more than 60% of the genomes being from Europe  (*n* = 373) and the United States of America (USA)  (*n* = 205). The Philippine genomes were found throughout the tree, indicating that the *P. aeruginosa* population captured in our survey largely represents the global diversity of this pathogen. Notably absent were the epidemic clones ST111 and ST175 (**Fig. 3A**), which, together with ST235, are responsible for MDR and XDR nosocomial infections worldwide.

**Figure 3A:**
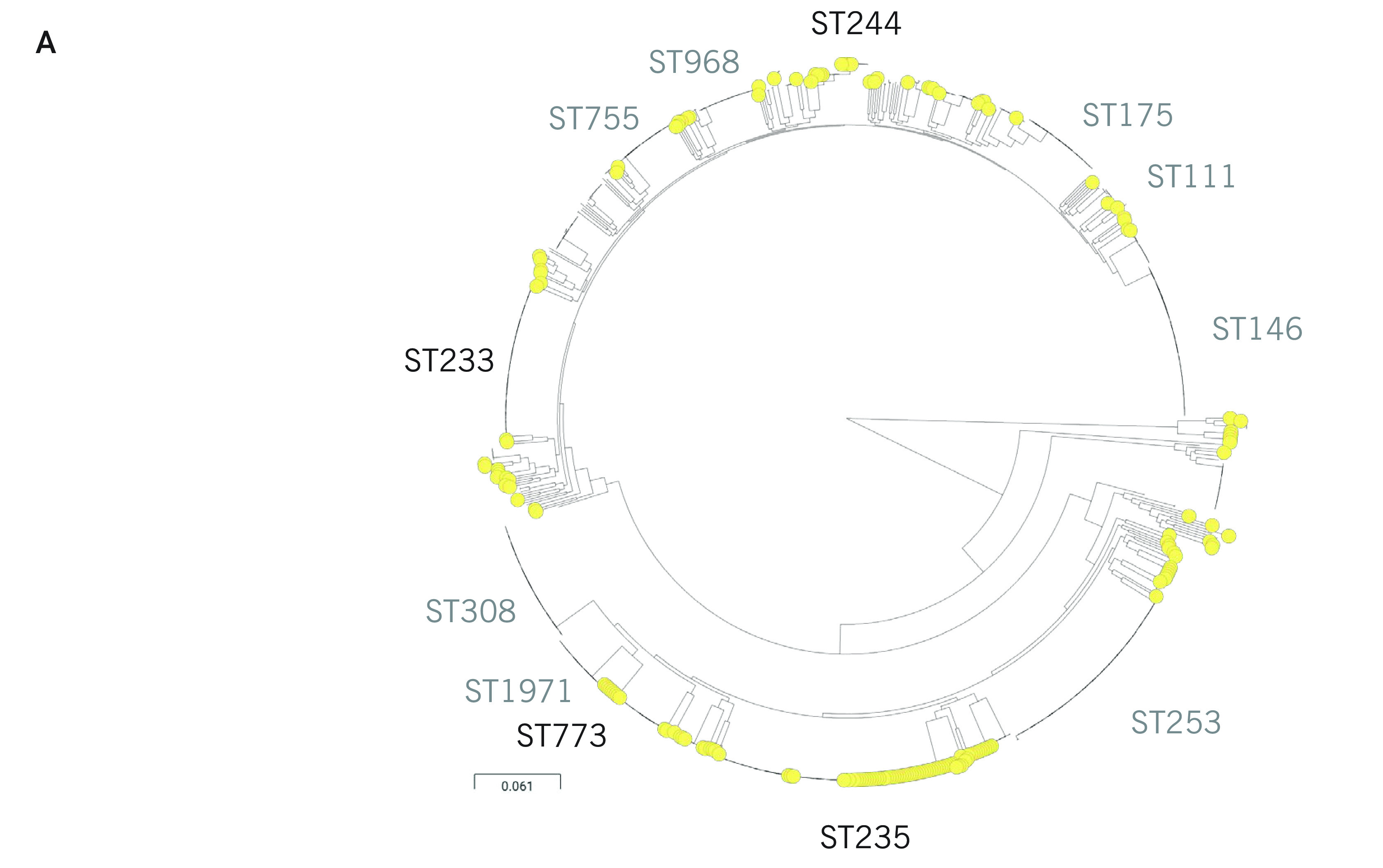
P. aeruginosa from the Philippines in the global context^a^

[**insert** Figure 3A]

A more detailed tree of 96 ST235 genomes of global distribution showed three major clades: clade 1 was represented by isolates from Japan, the Philippines, Poland and Thailand (*n* = 2); clade 2 showed the broadest geographic distribution across four continents and also included isolates from this study (*n* = 3); clade 3 comprised exclusively isolates from the Philippines  (*n* = 44, **Fig. 3B**), which raises the possibility that this lineage of ST235 is characteristic to the Philippines; however, introductions from the other globally dispersed lineages may also occur, as shown in clades 1 and 2.

**Figure 3B:**
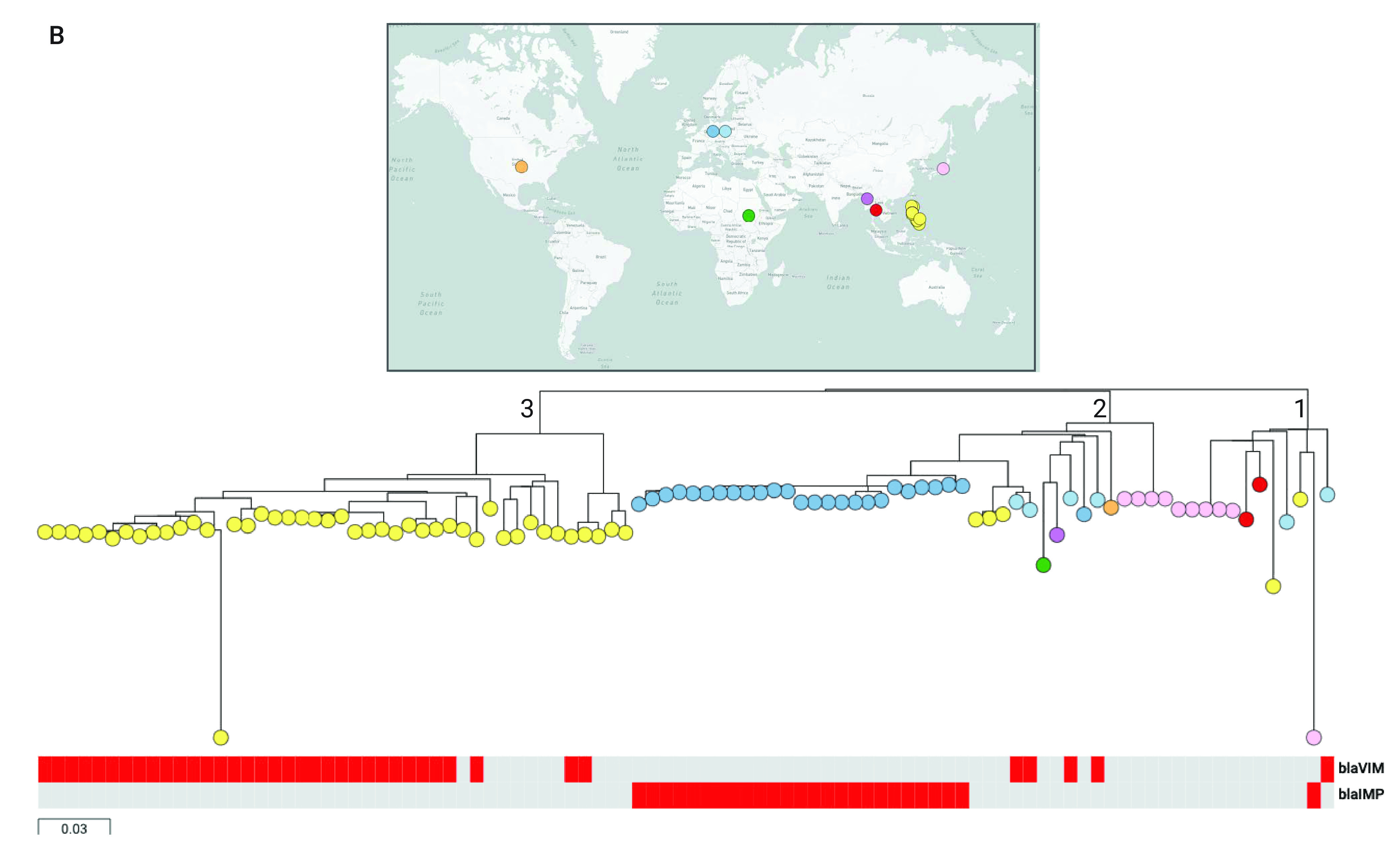
P. aeruginosa from the Philippines in the global context^a^

[**insert** Figure 3B]

## Discussion

In this study, we combined WGS and laboratory-based surveillance to characterize susceptible and resistant  *P. aeruginosa* circulating in the Philippines in 2013 and 2014, with a particular emphasis on resistance to carbapenems, which increased in the years preceding this survey. Drug-resistant *P. aeruginosa* infections are difficult to treat, resulting in poor patient outcomes. In a tertiary hospital in Manila, severity of illness and mortality rates were significantly higher among patients infected with drug-resistant *P. aeruginosa* than among those infected with susceptible isolates, while median duration of hospital stay was significantly longer. (*33*)

*P. aeruginosa* strains exhibit a complex interplay between resistance mechanisms, both intrinsic and acquired. (*34*) The current gaps in understanding of some of these mechanisms were reflected in the variable concordance between phenotypic and genotypic resistance for the different antibiotics, even for those antibiotics belonging to the same class (aminoglycosides). Our characterization of the carbapenem resistance showed a combination of diverse known mechanisms, from inhibition of antibiotic influx into the cell, to upregulation of antibiotic efflux out of the cell and carbapenemase enzymes. The concordance between phenotypic and genotypic predictions of AMR was high for the carbapenems, but it required a degree of curation of results that is not practical within public health settings.

There are clear limitations in the genomic predictions of AMR for *P. aeruginosa*. First, publicly available, curated databases are not comprehensive of all the known mechanisms. We found no mutations leading to upregulation of the chromosomal cephalosporinase AmpC (*bla*^PAO^), but an exhaustive search would require additional analyses. Second, the regulatory pathways of some mechanisms are not fully understood, such as those that regulate AmpC. (*34*, *35*) Third, extensive manual curation of some of the predictions is needed to ensure accuracy, for example of the loss-of-function mutations in the *oprD* gene.

The most prevalent clone in our data set was ST235 (27.8% of the isolates, *n* = 49), found throughout the Philippines. ST235 is a well characterized international epidemic clone causing drug-resistant nosocomial outbreaks. (*32*) Isolates carrying *bla*^VIM-2^ and belonging to ST235 were reported from Malaysia, the Republic of Korea and Thailand. (*13*) Using WGS, we showed evidence of potential localized hospital outbreaks of ST235, as well as of persistence or reintroduction of this clone within one hospital. The number of SNP differences between genomes of isolates from different patients (0–24) were consistent with those reported for a persistent outbreak of *P. aeruginosa* in a hospital in the United Kingdom of Great Britain and Northern Ireland. (*36*) We also showed evidence of transfer of ST235 between hospitals, with isolates from different hospitals separated by as few as seven SNPs. Patient transfer between hospitals is not common in the Philippines, but the sampling for this study only allows us to hypothesize about a possible role of the community, animals or the environment in the spread of this clone.

It was previously proposed that ST235 emerged in Europe around 1984, coinciding with the introduction of fluoroquinolones, and then disseminated to other regions via two independent lineages, acquiring resistance determinants to aminoglycosides and β-lactams locally. (*14*) Simultaneous acquisition of resistance to multiple antibiotics via integrons, transposons and integrative conjugative elements is well described in *P. aeruginosa*, (*36*) and is apparent in the distribution of resistance genes in our genomes. We have shown an example of a class 1 integron carrying six resistance genes in the genetic background of ST235. While this integron shared some features with others previously described in *P. aeruginosa*, (*13*, *32*) such as the 5¢ and 3¢ conserved segments, (*37*) the gene composition and synteny was different, supporting the hypothesis of local acquisition of resistance.

Country-specific ST235 lineages have been reported previously, (*11*, *14*) confirming that country-wide clonal expansions may occur in the context of the global circulation of this clone. A previous longitudinal study showed VIM-2-positive ST235 spreading throughout Belarus, Kazakhstan and the Russian Federation, albeit without the resolution of whole genome data. (*38*) The contextualization of our genomes with international ST235 genomes showed a distinct cluster of Philippine genomes with limited genetic variability, suggesting the clonal expansion and geographic dissemination of this lineage across the Philippines. Alternatively, this could be explained by the limited representation of the Western Pacific Region in the collection of global genomes, highlighting the need for public genome data with more even geographical coverage. Our retrospective survey contributed to bridging this gap by making raw sequence data available on public archives.

In conclusion, our detailed description of the epidemiology and resistance mechanisms of ST235 in the Philippines suggests that the burden of XDR *P. aeruginosa* infections in the Philippines may be largely driven by a local lineage of the international epidemic clone ST235. A recent study in a hospital in Jakarta, Indonesia analysed the population composition of *P. aeruginosa* before and after a multifaceted infection control intervention, with the relative abundance of ST235 almost halved in the 10 months post-intervention. (*39*) This highlights the importance of hospital infection control and of preventive measures to contain the spread of this high-risk clone.
